# SPITE VERSUS CHEATS: COMPETITION AMONG SOCIAL STRATEGIES SHAPES VIRULENCE IN *PSEUDOMONAS AERUGINOSA*

**DOI:** 10.1111/j.1558-5646.2012.01706.x

**Published:** 2012-11

**Authors:** R Fredrik Inglis, Sam P Brown, Angus Buckling

**Affiliations:** 1 Department of Zoology, University of OxfordOxford, OX1 3PS, United Kingdom; 2 Department of Environmental Sciences, Eidgenössiche Technische Hochschule ZürichCH-8092 Zürich, Switzerland; 3 Department of Environmental Microbiology, Swiss Federal Institute of Aquatic Science and Technology (Eawag)CH-8600 Dübendorf, Switzerland; 4 E-mail: fredrik.inglis@env.ethz.ch; 5 Centre for Immunity, Infection & Evolution, School of Biological Sciences, University of EdinburghUnited Kingdom; 6 Biosciences, University of ExeterPenryn, TR10 9EZ, United Kingdom

**Keywords:** Bacteriocin, kin selection, siderophore, social evolution, strong reciprocity, virulence

## Abstract

Social interactions have been shown to play an important role in bacterial evolution and virulence. The majority of empirical studies conducted have only considered social traits in isolation, yet numerous social traits, such as the production of spiteful bacteriocins (anticompetitor toxins) and iron-scavenging siderophores (a public good) by the opportunistic pathogen *Pseudomonas aeruginosa*, are frequently expressed simultaneously. Crucially, both bacteriocin production and siderophore cheating can be favored under the same competitive conditions, and we develop theory and carry out experiments to determine how the success of a bacteriocin-producing genotype is influenced by social cheating of susceptible competitors and the resultant impact on disease severity (virulence). Consistent with our theoretical predictions, we find that the spiteful genotype is favored at higher local frequencies when competing against public good cheats. Furthermore, the relationship between spite frequency and virulence is significantly altered when the spiteful genotype is competed against cheats compared with cooperators. These results confirm the ecological and evolutionary importance of considering multiple social traits simultaneously. Moreover, our results are consistent with recent theory regarding the invasion conditions for strong reciprocity (helping cooperators and harming noncooperators).

Social interactions are widespread in microbes and include behaviors such as communication, cooperative public goods production, and spiteful toxin production (West et al. 2007; Brown and Buckling 2008). There has been a recent proliferation of empirical and theoretical studies investigating the selective forces acting on social traits in bacteria (Bremermann and Pickering 1983; Frank, 1992,^1996^; West and Buckling 2003; Gardner et al. 2004; Massey et al. 2004; Harrison et al. 2006; Brown et al. 2008; Buckling and Brockhurst 2008; Vigneux et al. 2008; Inglis et al. 2009,^2011^; Köhler et al. 2009; Bashey et al. 2012). However, most studies to date consider social traits in isolation (but see Gardner et al. 2007; Lehmann et al. 2007; Harrison and Buckling 2009; Brown and Taylor 2010), yet multiple social traits are typically expressed simultaneously (Williams et al. 2007; Harrison and Buckling 2009). Here, we investigate how selection on two types of microbial social traits (spite: costly to both actor and recipient, and public goods cooperation: costly to the actor, beneficial to the recipient) can be altered by their interaction. Both spite and public goods cheating can be favored under the same competitive conditions, and we address how the success of a toxin-producing (spiteful) lineage is influenced by social cheating of toxin-susceptible competitors. We also investigate how competition between spiteful and cheating phenotypes affects disease severity (virulence). Both spite and public goods cooperation have been shown to be important factors in determining bacterial virulence as they both have an effect on population growth rate and density within a host (Harrison et al. 2006, Inglis et al. 2009, Köhler et al. 2009). Cooperative interactions have been shown to increase the virulence of infections (Harrison et al. 2006, Köhler et al. 2009) whereas spiteful interactions can attenuate virulence if the infecting population is also composed of individuals susceptible to the spiteful action (Massey et al. 2004, Vigneux et al. 2008, Inglis et al. 2009).

Spiteful behaviors appear to be common in microbes in the form of metabolically costly (and in some cases even suicidal) production of anticompetitor toxins (Riley and Wertz 2002; Hawlena et al. 2010). Spiteful behaviors can be favored by kin selection when nonrelatives are preferentially affected (negative relatedness between actor and recipient) (Hamilton 1970), and the adverse effect on the recipient is high relative to the cost to the actor. Differential effects of the toxins on nonkin versus kin is achieved by microbes via linkage between toxin and immunity genes, rendering all individuals with the same toxin gene resistant to the toxin; an example of a “green beard” (Hurst 1991; Gardner and West 2004, 2010; Massey et al. 2004; Greig and Travisano 2008; Wloch-Salamon et al. 2008; Inglis et al. 2009). Both theory and data suggest that selection for spite is maximized when the spiteful lineage is at an intermediate frequency in the population (Gardner and West 2004, Inglis et al. 2009). When the spiteful lineage is at low frequencies in the local interacting population, the benefit gained from the spiteful act (e.g., freeing up resources) will be more likely to be experienced by nonspiteful individuals. In contrast to this, at high local frequencies only a few competitors will be affected by the spiteful behavior, hence relative costs of spite will be high (Inglis et al. 2009).

Social environments that influence selection for spiteful behaviors are also likely to affect selection on indiscriminate cooperation. Indiscriminate cooperation (individually costly behaviors that benefit all others in the vicinity) is extremely common in microbes and includes the public goods production of extracellular enzymes and nutrient-scavenging molecules, such as siderophores (Buckling and Brockhurst 2008). Maintaining cooperation in a population relies on individuals interacting with their relatives, which are more likely to possess the same cooperation gene, due either to kin discrimination or population viscosity (Hamilton 1963; Smith 1964). Otherwise “social cheats,” individuals that do not pay the cost of the cooperative behavior but reap all the rewards, are able to invade and displace the cooperators. Note that spiteful behaviors can also be viewed as a form of indirect, discriminating altruism, in that unaffected individuals with the spite allele benefit from the behavior through removal of competitors (Lehmann et al. 2006).

In this study, we take a joint theoretical and empirical approach, using the pathogenic bacterium *Pseudomonas aeruginosa*, to determine how the success of spiteful cooperators is affected by whether the susceptible competitors are themselves public goods cheats or cooperators. Both strategies exist in natural populations of *P. aeruginosa* (e.g., Bodilis et al. 2009), yet to date selection on spite and selection on cooperation have only been studied in isolation (i.e., spite vs. nonspite; cooperators vs. cheats). We focus on the production of pyocin S2 and type 1 pyoverdine by *P. aeruginosa*, a spiteful and a public goods cooperative trait, respectively. Pyocin S2 is a protein-based toxin that inhibits phospholipid synthesis through DNA breakdown, whereas type 1 pyoverdine is a fluorescent yellow–green, high-affinity iron chelator (a siderophore). We also investigate the impact of competition between spiteful and cheating genotypes on *P. aeruginosa* virulence in an insect model system.

## Materials and Methods

### BACTERIAL STRAINS

*Pseudomonas aeruginosa* strain PAO1 was used as the spiteful cooperator as it is a known producer of pyocin S2 (a bacteriocin) and pyoverdine type 1 (a siderophore; Meyer 2000; Denayer et al. 2007; Inglis et al. 2009). PAO 1150–2, a transposon, knock-out mutant of the pyocin S2 gene, *psy2*, that still produces pyoverdine type 1 was used as an isogenic nonspiteful cooperator. These strains were competed against an S2-susceptible strain, O:9, that does not produce any known toxins that act against PAO1 or PAO 1150–2, but does produce equivalent levels of pyoverdine type 1. We also competed the two strains against mutants of O:9 that showed marked reductions in pyoverdine production. These mutants were generated by evolving O:9 for approximately 14 generations in iron-limited conditions (see below) in batch culture (transferred daily 1 in 100 dilution into fresh media for two days). Six phenotypically white colonies of O:9 that exhibited a reduction in iron-chelating compounds of more than 60% (as confirmed by a CAS assay; Harrison et al. 2006) were chosen. Pyocin sensitivity of the six O:9 siderophore “cheats” was confirmed using a simple plate assay as described in Inglis et al. (2009). A detailed description of strains used can be found in Table 1.

**Table 1:** A list of strains used in the experiments with details concerning pyoverdine and pyocin production and susceptibility to pyocin S2.

Strains used	Description	Pyocin S2 production	Pyocin S2 sensitivity	Pyoverdine production
PAO1	Wild-type *P. aeruginosa* ATCC 15692	Yes	No	Yes
PAO1150-2	Transposon Knockout Mutant of *P. aeruginosa* PW3083 (Jacobs et al. 2003)	No	No	Yes
O:9	Wild-type *P. aeruginosa*, pyocin S2 sensitive (Smith et al. 1992)	No	Yes	Yes
O:9 mutant 3	″	No	Yes	No
O:9 mutant 4	″	No	Yes	No
O:9 mutant 6	″	No	Yes	No
O:9 mutant 40	″	No	Yes	No
O:9 mutant 50	″	No	Yes	No

Growth rates for the ancestral O:9 and evolved mutants were calculated (using approximate Malthusian parameters; see competition assays described below) when the strains were growing in monoculture in iron-supplemented media and found to be the same (*t* = 0.273, 11 df, *P* > 0.79). However, the evolved mutants showed a decrease in growth rate when grown in monoculture in iron-limited media, compared to the ancestral strain (*t* = 4.84, 11 df, *P* < 0.0047). The evolved mutants also showed increased growth rate in iron-limited media when grown with a pyoverdine type 1 producing strain (PAO1150–2, *t* = 2.69, 11 df, *P* < 0.0227) compared to when grown in isolation, indicating they are able to use type 1 pyoverdine. The evolved mutants reduced the growth rate of the pyoverdine-producing strain (PAO1150–2, *t*= 3.1, 11 df, *P* < 0.021) compared to when PAO1150–2 is grown in isolation, indicating that this interaction can be considered “cheating.” Growth rates of PAO1 and PAO1150–2 were also compared when grown in monoculture and did not differ significantly (*t*= 0.649, 11 df, *P* > 0.73).

### COMPETITION ASSAYS

Overnight cultures of all strains were grown in 30-mL glass universals containing 6 mL of King’s medium B (KB), shaking at 0.65 *g* (relative centrifugal force) and 37°C for 18 h, and then diluted to an OD_600nm_ of 1.8 (approximately cell density = 10^9^/mL) to ensure similar numbers of bacteria per milliliter. These cultures were subsequently grown on agar plates to determine the number of bacteria inoculated at the beginning of the experiment, using colony-forming units (CFUs) as an approximate measure. Thirty-milliliter glass universals containing 6 mL of KB broth were inoculated with a total of 10^4^ cells with different starting frequencies of the individual strains. Populations were subjected to iron-limited conditions by the addition of 100 μg/mL human apotransferrin (Sigma, Gillingham, UK), a natural iron chelator, and 20 mM sodium bicarbonate, necessary for iron chelator activity (Meyer et al. 1996).

Our approach was to compare the relative fitness of PAO1 and the isogenic pyocin knockout when competing at different frequencies against the pyocin-susceptible pyoverdin producer (cooperator) and the pyocin-susceptible pyoverdin “cheats.” Specifically, we competed PAO1 against each of the six O:9 isolates exhibiting reduced siderophore production at starting frequencies of 99.9%, 99%, 90%, 50%, 10%, 1%, and 0.1%, and separately against the O:9 ancestor in six replicates. The same competition experiments were carried out for PAO1150–2 against the O:9 cooperators and cheats. Cultures were propagated in a shaking incubator at 0.65 *g* and 37°C and sampled after 96 h, allowing time for effects of the bacteriocin to be observed (Inglis et al. 2009). We plated the various treatments on KB agar plates and counted the number of CFUs for each strain: all strains were easily distinguishable from one another because of unique colony morphology and size. At the more extreme frequencies, antibiotic plates were required to give better resolution of colony counts, which was possible due to the different antibiotic resistance profiles of the assorted strains (PAO1 resistant to streptomycin 1250 μg/mL, O:9 resistant to rifampicin 312.5 μg/mL, and PAO 1150-2 resistant to tetracycline 312.5 μg/mL). Selection coefficients (*S*) were used to estimate the fitness of PAO1 and PAO1150–2 at a range of starting frequencies relative to the common competitors O:9 and O:9 siderophore cheats, where *S =* (*m*_PAO1/1150-2_–*m*_O:9_)/*m* _O:9_, and (*m*) refers to ln(final density/starting density) (Lenski et al. 1991). Selection coefficients were preferable to simply using growth rates (*m*), to control for between-tube variation.

### IN VIVO VIRULENCE ASSAYS

Virulence assays were performed as previously described (Miyata et al. 2003; Harrison et al. 2006; Inglis et al. 2009). Briefly, overnight cultures of PAO1, the six O:9 isolates, and PAO1150–2 were diluted in minimal salt solution. Fifth instar waxmoth (*Galleria mellonella*) larvae (Livefood UK, Rooks Bridge, UK; http://www.livefood.co.uk) were randomly allocated to be inoculated with 10^4^ CFUs of PAO1/O:9 and PAO 1150–2/O:9 mixtures. The starting frequencies of the bacterial combinations consisted of 99%, 50%, and 1% PAO1 to O:9 or PAO1150–2 to O:9. Larvae were swabbed with 70% ethanol to prevent contamination of the injection site and injected into the abdomen using Terumo 1-mL disposal syringes and BD Microlance 30G ½″ needles. The injection volume was 50 μl in all cases. Twenty larvae were assigned to each treatment, and a further 20 larvae were injected with 50 μl of minimal salt solution as negative controls. Larvae were then incubated at 37°C and monitored for death at 30-min intervals between 10 and 18 h postinoculation. Larvae were scored as dead if they failed to respond to mechanical stimulation of the head.

Overall density of the different bacterial strains within the caterpillar hosts was also measured. Caterpillars were inoculated as previously described (Harrison et al. 2006; Inglis et al. 2009) and incubated for 8 h at 37°C. Larvae were then weighed, dipped in 70% ethanol to kill surface contaminants, and homogenized in 500 μl minimal salt solution using a plastic pestle. Homogenates were centrifuged at 3000 rpm for 3 min to pellet the solid, and aliquots of diluted homogenate plated onto KB agar. Agar plates were supplemented with 15 μg/mL ampicillin to select against growth of native larval-gut bacteria (this concentration of ampicillin does not affect the growth of *P. aeruginosa*) (Harrison et al. 2006). Plates were incubated overnight at 37°C and subsequently scored for CFUs. All statistical analyses were performed in R (2.9.2).

## Results

### MODEL

We extend a simple mathematical model for the evolution of bacterial spite (Inglis et al. 2009) to address whether the invasion of a spiteful mutant will be enhanced if the mutant also invests more than the resident in public goods cooperation (i.e., if the spiteful lineage is competing against a public goods cheat). The heuristic model framework in Inglis et al. 2009 (building on Gardner et al. 2004 and Frank 1998) assumes that the bacterial population is structured into patches (e.g., hosts), and that the fitness of the focal mutant strain (present at a frequency *p* within the focal patch, the remaining 1–*p* being the resident strain) is dependent on its growth rate measured relative to its competitors, a fraction *a* of which is found locally within the patch and a fraction 1–*a* is found globally across the population. Further details on the model structure are presented in the Appendix.

**Figure 1:**
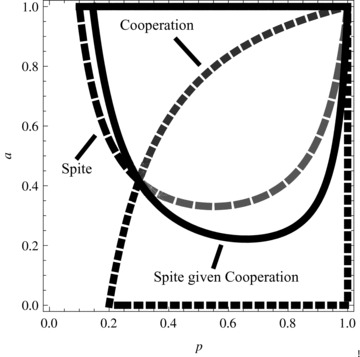
Mutant invasion conditions as a function of local competition *a*, and local frequency, *p.* Regions favoring investment in spite only (dashed line, eq. A5), cooperation only (dotted line, eq. A7), and spite given cooperation (solid line, eq. A8). Models are derived in the Appendix. Parameters are *c =* 0.4, *d*= 0.2, *k =* 2, *b =* 2.

Figure 1 illustrates the established invasion conditions for mutant strains investing only in spite (dashed-line region) or only in cooperation (dotted-line region) as a function of the scale of competition (from purely local, *a* = 1, to purely global, *a* = 0) and local frequency, *p.* In keeping with existing theoretical and empirical findings, we see that spite is most favored under conditions of high local competition and intermediate local frequencies (dashed-line region [Gardner et al. 2004; Gardner and West 2004; Inglis et al. 2009]), whereas cooperation is most favored under conditions of low local competition and high local frequencies (dotted-line area [Frank 1998; Griffin et al. 2004]). Note that if we assume the individual trait to be vanishingly rare across the global population, and locally present at frequency *p*, then *p* is the coefficient of relatedness measured with respect to the global population (Frank 1998; Inglis et al. 2009).

**Figure 2:**
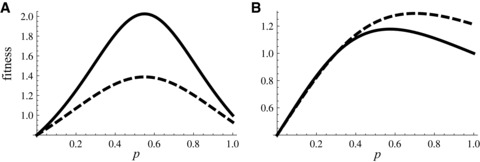
Fitness of spiteful strains as a function of initial frequency *p* and scale of competition *a*. (A) Fitness of spiteful strain competing against a sensitive strain, no difference in cooperative investments (eq. A4). Solid line = purely local competition (*a*= 1), dotted line = largely local competition (*a =* 0.7). Parameters are *c =* 0.4, *d*= 0.2, *k =* 2, *b =* 2. (B) Fitness of spiteful cooperator competing against sensitive cheat (eq. A3). Solid lines, *a*= 1. dotted line, *a*= 0.7. Parameters are *c =* 0.4, *d*= 0.2, *k =* 2, *b =* 2.

In Figure 2, we focus on the fitness of a spiteful strain under conditions of significant local competition (high *a*). In Figure 2A, we see again the established result that spiteful versus sensitive competition in the absence of any cooperative differential leads to a peak in spite fitness at intermediate frequencies (Gardner et al. 2004, Inglis et al. 2009). In Figure 2B, we see how this picture changes when we introduce a cooperative differential, specifically when the sensitive strain is cheating on the public goods provision of its spiteful neighbor. The result is a shift in peak spite (plus cooperation) fitness toward higher frequencies. Below we experimentally test these predictions, but first we ask whether this pattern can be understood simply as an additive combination of a spite fitness effect (fitness maximized at intermediate frequencies) and a cooperation effect (fitness maximized at high frequencies), or whether the value of spiteful investments changes as a result of association with cooperative investments.

Using our extension to the Inglis 2009 model (Appendix) we ask, what is the effect of increasing cooperation on the value of investments into spite? We find that additional investment into cooperation acts to change the relative costs and benefits of investments into spite in a systematic way. Specifically, selection for spite is enhanced whenever the conditions for investment in cooperation alone are met (dotted-line area in Fig. 1). Conversely, selection for spite is inhibited if cooperation is selected against. Thus, selection for spite (given cooperation) is enhanced at high local frequencies but diminished at low local frequencies. These results have a simple biological interpretation: when a cooperative focal strain is locally prevalent (high *p*) then local population growth rate will be high, and hence the relative growth rate reduction of an additional fixed investment into spite is reduced. This expands the region of favorable investments into spite toward higher frequencies (Fig. 1).

The heuristic approach underlying Figure 1 (Frank 1998; Gardner et al. 2004; Inglis et al. 2009) assumes that investments in spite and/or cooperation have negligible consequences for the local frequency *p* of the focal strain (weak selection). By relaxing this assumption, we can see further reasons to expect a strengthening of selection for spite at higher frequencies. Specifically, if we assume that cooperation reduces the local frequency *p* (due to cheater exploitation), then when *p* is initially high (above the point of peak investment in spite, see Appendix), any reduction in *p* toward intermediate frequencies will generate an increased reward from spiteful investments.

**Figure 3:**
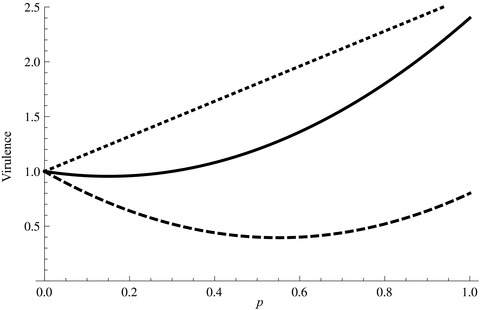
Virulence (mean within-host growth rate) as a function of frequency of focal strain. For all lines, the resident strain displays neither social trait, and has virulence = 1. Dotted line = a focal cooperative strain. Dashed line = a focal spiteful strain. Solid line = a focal cooperative and spiteful strain. Other parameters, *b =* 2, *c =* 0.4, *d =* 0.2, *k =* 2.

Finally, we ask what are the implications for pathogen virulence of the coupling of cooperative and spiteful traits in a single lineage? Looking at each trait in isolation, we recover established results (Fig. 3, Appendix), specifically that the virulence of infections containing cooperative genotypes is maximized when cooperative genotypes are at high frequencies (Brown et al. 2002; West and Buckling 2003; Harrison et al. 2006; Brown et al. 2009), whereas the virulence of infections containing spiteful (and susceptible) genotypes is minimized when spiteful genotypes are at intermediate frequencies (Gardner et al. 2004; Inglis et al. 2009). Turning to the spiteful cooperators, we find that virulence will follow a hybrid path with increasing local frequency of the spiteful cooperator *p* (solid line, Fig. 3), with the intermediate minima (characteristic of spiteful interactions) lost whenever investments in cooperation are sufficiently valuable, relative to the value of investments in spite.

### IN VITRO

Our model predicts that competing against less-cooperative public good “cheats” should boost the relative fitness of invading spiteful versus nonspiteful genotypes at higher local frequencies, *p*. This prediction holds across a wide range of scales of increasingly local competition. To empirically test this hypothesis, we determined the relative growth rate of a cooperating spiteful strain when competed against both cheating and cooperating sensitive strains at a range of starting frequencies. These treatments test the relationship between starting frequency and fitness for spite only (dashed line in Figs. 1, 2A) and spite given cooperation (solid line in Figs. 1, 2B) in our heuristic model. We also determined the growth rate of an isogenic cooperating but nonspiteful strain when competing against the same susceptible cheats and cooperators as above: the former examines the relationship between cooperation-only fitness and starting frequency (dotted line in Fig. 1), whereas the latter is a no-social-conflict control (no spite and everyone cooperating), where no relationship was expected. Competitions were carried out under local competition (single shaken tubes), although the scale of competition was less than entirely local (i.e., *a* < 1), because wall-growth inevitably creates a degree of spatial structure within each tube.

**Figure 4:**
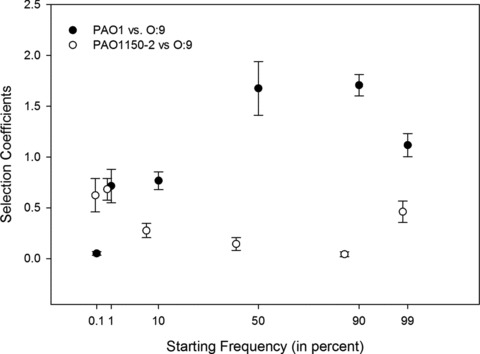
Relative growth of spiteful compared to nonspiteful bacteria when competed against sensitive cooperators (i.e., all strains produce siderophores) in iron-limited media. Spiteful bacteriocin production (PAO1: siderophore and bacteriocin producer vs. O:9: siderophore producer sensitive to bacteriocin) is favored at intermediate starting frequencies as previously described in noniron-limited media. Error bars represent ± 1 SE of the mean.

We calculated selection coefficients for the spiteful and nonspiteful cooperator under the different scenarios. We first determined how the relative benefit of spite changed with frequency when competing against sensitive genotypes that were also producing wild-type levels of siderophore in iron-limited media (i.e., no differential investment in cooperation). This experiment replicates our previous study (Inglis et al. 2009), except here the media is iron-limited. Consistent with this previous work and theoretical predictions, the benefit of spite was maximized at intermediate frequencies (linear term *F*_1, 31_= 44.65, *P* < 0.001; quadratic term *F* _1, 30_= 29.14, *P* < 0.001) (Fig. 4). By contrast, the nonspiteful strain displayed a weakly negative relationship with starting frequency (slope =–0.22, linear term *F*_1, 33_= 5.63, *P* < 0.024). This negative relationship was not predicted by our model as there is no obvious social conflict, and may have arisen because competing strains were not isogenic, and are perhaps using slightly different resources, resulting in negative frequency dependent fitness (Inglis et al. 2009).

**Figure 5:**
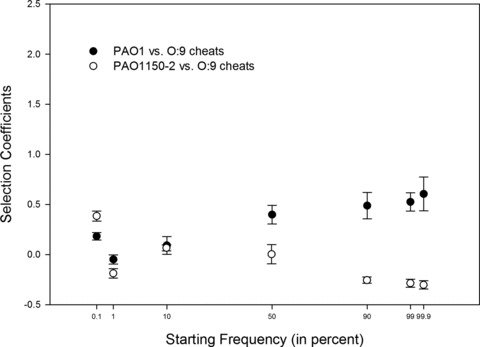
Relative growth of spiteful compared to nonspiteful bacteria when competed against sensitive public goods cheats (i.e., bacteria that do not produce siderophores). Spiteful bacteriocin production (PAO1: siderophore and bacteriocin producer vs. O:9 cheats: siderophore nonproducer sensitive to bacteriocin) is now favored at intermediate and high starting frequencies, whereas cooperators (PAO1150–2: siderophore producer vs. O:9 cheats: siderophore nonproducer) display a strong negative frequency dependent relationship with starting frequency. Error bars represent ± 1 SE of the mean.

Next we competed the spiteful genotype against the sensitive genotypes that display a marked reduction in siderophore production (sensitive cheats). Spite was favored at intermediate and high starting frequencies (a clear peak shift to the right), as predicted by our model (linear term *F*_1, 39_= 31.19, *P* < 0.001; quadratic term no longer significant) (Fig. 5). This difference between the spiteful “cooperators” when competed against sensitive cheats or sensitive cooperators was highly significant when a single polynomial model was fitted for the whole dataset (*x*-intercept: *F*_1, 73_= 111.56, *P* < 0.001, difference between slopes: *F*_1, 73_= 7.66, *P* < 0.007). When the isogenic nonspiteful cooperators were competed against the sensitive cheats, we still observed a negative relationship between starting frequency and selection coefficients (slope =–0.29, linear term *F*_1, 40_= 33.23, *P* < 0.001). However, the fitness of the nonspiteful cooperators was lower when competing against sensitive cheats compared to sensitive cooperators (*F*_1, 74_= 49.55, *P* < 0.001). This difference is attributable to the growth rate advantage sensitive cheats have over sensitive cooperators in the presence of exogenously produced siderophores.

### IN VIVO

**Figure 6:**
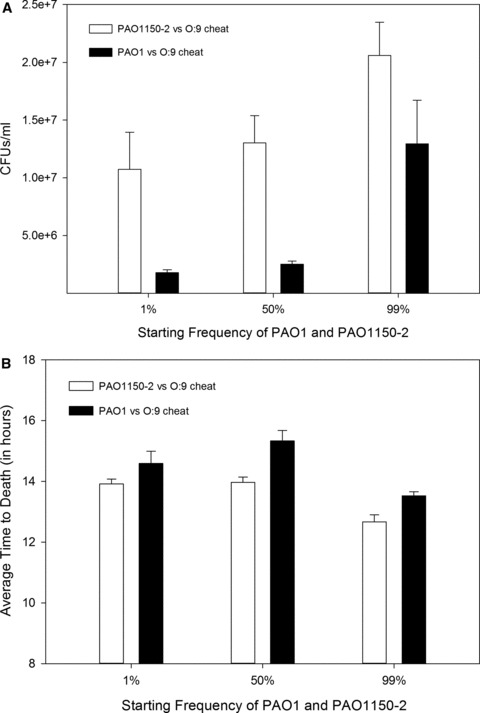
Bacterial density and virulence in a caterpillar host. (A) Bacterial density affected by starting frequency of spiteful and nonspiteful cooperators. Lowest densities were observed in low and intermediate spiteful cooperator treatments. High starting frequencies of spiteful cooperators reached similar densities to that of low and intermediate nonspiteful cooperators. The highest density was observed in the high nonspiteful cooperator frequency. Error bars represent ± 1 SE of the mean. (B) Average time to death of caterpillars infected with different starting frequencies of spiteful and nonspiteful cooperators. Lowest virulence (as measured by time to death) was observed in the low and intermediate spiteful cooperator treatments. The highest virulence is found in the high starting frequency of nonspiteful cooperator, with similar virulence occurring in the intermediate and low nonspiteful cooperator treatments and high starting frequencies of spiteful cooperators.

We next determined the importance of spiteful interactions in driving the relationship between starting frequency of competing genotypes and virulence, when cooperating genotypes compete with cheats. We manipulated starting populations to give high (99%), intermediate (50%), and low (1%) frequencies of the spiteful cooperator (PAO1) relative to the susceptible cheats (O:9 siderophore mutants), and compared these results with those obtained for coinfections with the nonspiteful cooperator (PAO1150–2) and susceptible cheats (O:9 siderophore mutants). Our model suggests the reduction in virulence associated with intermediate frequencies of a spiteful genotype (as seen in both theoretical and empirical studies; Inglis et al. 2009) should be attenuated or removed when spiteful cooperators compete against cheats. Virulence models (including those cited above) typically assume that virulence is simply a positive function of within-host growth rate, and when we measured density of bacteria within caterpillars, our results were entirely consistent with our model prediction. Both cooperative strains (spiteful/nonspiteful) displayed a positive curvilinear relationship between starting frequency and bacterial density after 8 h growth when in competition with susceptible cheats (linear term *F*_1, 95_= 43.15, *P* < 0.0001; quadratic term *F*_1, 93_= 4.65, *P* < 0.034), with nonspiteful cooperators reaching overall higher densities (*F*_1, 94_= 75.56, *P* < 0.0001) (Fig. 6A). This positive monotonic relationship between in vivo growth rate and cooperator frequency is consistent with theory (including our model) (Brown et al. 2002; West and Buckling 2003) and experimental results (Harrison et al. 2006). Crucially, there was no evidence of reduced density at intermediate frequencies of spiteful cooperators, as has been observed under almost identical experimental conditions when the spiteful cooperator competed against the susceptible cooperator used in this study (Inglis et al. 2009).

We also measured virulence directly, in terms of time to death of caterpillars. These results were broadly consistent with the model and in vivo growth rate data. We found that virulence was most greatly attenuated at intermediate and low frequencies of spiteful cooperators in competition with sensitive cheats (frequency was highly significant in the survival analysis, *P* < 0.0001, with 50 df and intermediate and low frequencies differed significantly from the high frequency, *P* < 0.004 and *P* < 0.001 respectively, but there was no significant difference between intermediate and low frequencies, *P* > 0.075 with 51 df) (Fig. 6B). The highest virulence was observed at high frequencies of nonspiteful cooperators, and no difference was seen between intermediate and low frequencies (frequency was again highly significant in explaining survival, *P* < 0.0001 with 50 df, but there was no significant difference between intermediate and low frequencies, *P* > 0.53 with 51 df) (Fig. 6B).

## Discussion

We investigated how the success of a siderophore- and bacteriocin-producing *P. aeruginosa* genotype is altered by whether its conspecific competitor is itself a siderophore producer or cheat, and the resulting impact on virulence in insects. We show in vitro that the unimodal relationship between the fitness of spiteful, toxin-producing bacteria and their frequency (Fig. 4) is altered when the spiteful genotype is more cooperative than its competitor, resulting in a systematic shift in peak fitness toward higher frequencies (Fig. 5). Furthermore, we demonstrate that the virulence of infections of insects where there is competition between cooperating and cheating bacteria is significantly altered depending on the starting frequencies of the genotypes, and whether the cooperating bacteria are also spiteful (Fig. 6A, B). We found positive monotonic relationships between virulence and cooperator frequency for both spiteful and nonspiteful genotypes, with greater, overall virulence in infections with nonspiteful bacteria. Importantly, previous work has shown that in the absence of a cooperator–cheat conflict, virulence is minimized at intermediate frequencies of spiteful genotypes (Inglis et al. 2009), demonstrating that competing against social cheats fundamentally alters the relationship between virulence and spite. All these results are qualitatively consistent with the predictions from our mathematical model (Figs. 1–3, Appendix).

Our model gives us some very simple insights as to why spite is more beneficial at high frequencies when competing with public goods cheats. First, the model shows that simultaneous investments in cooperation can change the relative benefit/cost ratio of investments into spite, with the result that selection for spite is systematically enhanced when spiteful cooperators are at high frequencies. This is because high frequencies of cooperation will increase the growth rate of the focal strain, and so lessen the relative growth rate costs of a fixed investment into spite. Second, if we additionally allow for cheats to initially increase locally in frequency, due to their competitive advantage with respect to the public good trait, then investments in spite can be further favored if the initial frequency of the spiteful cooperators is sufficiently high. This is because spiteful behaviors are maximally favored at intermediate frequencies, and the invasion of the cheats can push the frequency of the spiteful cooperators from high to intermediate frequencies. The net result of both processes is to shift the maximal value of spite to higher frequencies. There may of course be other mechanisms operating that help to explain our results that are not captured in our model: for example, if these traits are expressed at different times. However, it is not necessary to invoke such specific biological details to explain the results.

We wish to reiterate that that our model is not a demographically explicit or “closed” model of evolutionary change, instead we take a simplifying “black box” approach by summarizing numerous demographic effects via the parameters *a* and *p.* Although the heuristic “open model” approach sacrifices ecological specificity, it gains in terms of generality (allowing comparisons across diverse demographies) and tractability (see e.g., Gardner and West 2006; Brown and Taylor 2010). Clearly, the two approaches are highly complementary, and it remains to be seen what further insights can be generated via closed models of multiple social trait evolution under specific demographic regimes.

Although our experimental results highlight how public goods cooperation enhances the value of spite, our theory also illustrates that the converse can also hold true—spite can enhance the value of public goods investment by increasing the local frequency of producers (eq. A10). Together, these results outline a potential mechanistic synergy between spiteful and cooperative traits, which suggests that the long-term joint evolutionary dynamics will be more complex than predicted by analysis of either trait alone (Brown and Taylor 2010).

Qualitative patterns in our virulence data were consistent with previous studies examining social traits in isolation (Brown et al. 2002; West and Buckling 2003; Gardner et al. 2004; Harrison et al. 2006; Inglis et al. 2009). First, virulence, as determined by the growth rate of the whole infecting population, increased with the frequency of siderophore cooperators. This is unsurprising, as siderophore concentration increases growth rate by allowing iron to be scavenged from the caterpillar host. Second, the presence of spiteful genotypes reduced mean in vivo growth rate and virulence, because bacteriocin production is both costly to the actors (cell lysis) and the victims (arrests cell replication by inhibiting phospholipid synthesis). However, in contrast to previous work investigating spite and virulence in *P. aeruginosa* (Inglis et al. 2009) in the absence of a cooperator–cheat conflict, virulence was not minimized at intermediate frequencies of spiteful genotypes, but rather was minimal at both low and intermediate frequencies and increased at high frequencies. This is the pattern expected from our theoretical work, and is presumably the result of increased conflict between spiteful and cheating strains at low and intermediate frequencies. When the spiteful lineage is at low frequency in the competing population, there will be a reduction in growth rate as siderophore levels are low, thereby reducing virulence. At intermediate frequencies of the spiteful genotype, siderophore production will also be reduced to some extent, and total bacterial density reductions resulting from killing the sensitive cheats will also be at their greatest. The time to death data were largely consistent with the in vivo growth data, with variation likely to be attributable to the genetic heterogeneity of the caterpillars.

In the current study, cheating genotypes are always susceptible to pyocins, and hence spite can be considered as a form of punishment in this experimental context; an association noted in some recent theoretical studies (Nakamaru and Iwasa 2006; Lehmann et al. 2007). The concept of strong reciprocity—the punishment of noncooperators by cooperators—is suggested to be an important process in the evolution of cooperation (e.g., Bowles and Gintis 2004). Our theoretical results suggest that punishment can be favored over nonpunishment when at intermediate to high frequencies in structured populations, but cannot invade from rare. This result is broadly consistent with recent theory under conditions where cooperation and punishment are not in linkage; an assumption appropriate for our theoretical results, given that we are measuring fitness of cooperating punishers invading cooperating nonpunishers. By contrast, punishment can theoretically invade from rare when these traits are in linkage (Lehmann et al. 2007). It is interesting to note that there is an association between pyocin and pyoverdine outside of this experimental context. Specifically, *P. aeruginosa* pyocin S2 is taken up through the primary pyoverdine receptor, FvpA type 1 (Denayer et al. 2007). Genotypes that evolve to exploit a strain’s pyoverdine type (of which there are a number of alleles [Tummler and Cornelis 2005]) may, therefore, become vulnerable to the competitor’s pyocin.

Here, we have shown that spiteful behaviors, or more specifically bacteriocin (pyocin) production, are critically affected by simultaneous investments in another social trait, in this case indiscriminate public goods cooperation (pyoverdine production). We have shown that this, in turn, can have important repercussions when considering virulence in *P. aeruginosa* infections. Both pyocin (spite) and pyoverdine (cooperation) are likely to be of importance in clinical settings, especially cystic fibrosis, where pyocin-producing and pyoverdine-cheating strains are commonly found during the course of an infection (Govan 1986; De Vos et al. 2001). This study may, therefore, help to explain patterns of virulence in natural infections, and could even have practical implications in terms of manipulating the competitive arena through some type of directed therapy (i.e., the introduction of pyocin-producing, public goods cheats), thereby reducing overall virulence. More generally, spiteful and cooperative behaviors are almost ubiquitous among bacteria (and may be common among other microbes, both pathogenic and nonpathogenic), hence an understanding of the evolution of these behaviors may be key to understanding microbial community dynamics and functions.

## 

Associate Editor: J. Strassmann

## Appendix

We base our approach on Frank’s (Frank 1998) model of evolution under local competition, where fitness *w* is defined by the fecundity of a focal strain *g_f_*, measured relative to the focal strain’s competitors—a fraction *a* of which is found locally within a focal patch (with average fecundity *g_l_*) and a fraction (1–*a*) of which is found globally across the population (with average fecundity *g_g_*), that is,

(A1)

Following Inglis et al. 2009 and Gardner et al. 2004, we define focal, local, and global growth rates as a function of independent investments by the focal mutant strain in spite (and now, cooperation), in a population of residents that do not invest in either trait. Note that our analyses allow for mutants that differentially invest in spite only (recovering results of Inglis 2009), cooperation only (recovering Frank 1998, p. 115), or both.

Investments in spite and in cooperation incur costs expressed as reductions in growth rate of *d* and *c*, respectively (relative to a normalized growth rate of 1 for an asocial clonal population). We further assume that the focal strain is immune to its bacteriocin, but a proportion *pk* cells of the competitor strain is killed, thus *k* measures the killing efficiency of the bacteriocin produced by the killer lineage, present in proportion *p.* The cooperative trait in turn renders a growth advantage *pb* to all cells within the patch, regardless of their strain type. Therefore, we have

(A2a)

(A2b)

(A2c)

Note that the advantage to a focal lineage of spiteful investments is felt via a reduction in the fecundity of their local competitors, *g_l_.* Together, these assumptions yield a neighbor-modulated fitness function

(A3)

### SELECTION ON SEPARATE INVESTMENTS IN COOPERATION AND SPITE

We begin by considering the growth of a rare mutant deviant in one strategy only, invading a resident population. First, we consider the fitness of a mutant differing only in its spiteful investment (*w_s_*) setting *b = c =* 0 in equation (A3) and so recovering the fitness function of Inglis 2009,

(A4)

The condition for the focal spiteful strain to be favored is when its growth rate exceeds that of its competitors, that is, when *w_s_* > 1 (dashed-line region in Fig. 1), which simplifies to

(A5)

This is an established result (Gardner et al. 2004, Inglis et al. 2009, empirically supported by Inglis et al. 2009), which can be expressed in terms of Hamilton’s rule, *RB* > *C* with negative “benefits” and negative relatedness to the victims of the spiteful act (*C_s_= d*, *B_s_=–*(1 –*p*)*k*, *R_s_=*–*ap*/(1 –*ap*)). Note that here relatedness to victims is measured relative to the pool of competitors (Queller 1994), as opposed to being measured relative to the entire population.

Next, we can take the same approach to investigate selection on mutants differing only in their cooperative investments. We consider a mutant cooperative strain by setting *k = d =* 0 in equation (A3),

(A6)

The condition for the focal cooperative strain to be favored (*w_c_* > 1) simplifies to

(A7)

illustrated by the dotted-line region in Figure 1. This again is an established result (Frank 1998, pp. 114–115, empirically supported by Griffin et al 2004), which can be expressed in terms of Hamilton’s rule, *RB* > *C*, with *C_y_= c*, *B_y_= b*, and *R_y_=* (1 –*a*)*p*/(1 –*ap*), which is again a measure of relatedness relative to competitors (Queller 1994), but now relatedness to clone mates, relative to competitors. Note that in the limit of the focal strain being globally rare, relatedness to clone mates, measured relative to the global population, remains *R = p* (Inglis 2009, Gardner 2004).

### SELECTION ON SPITE, GIVEN SIMULTANEOUS INVESTMENTS IN COOPERATION

Next we ask whether mutant investments in cooperation will help or hinder investments in mutant spite. To address whether additional investments in spite are beneficial, on a background of mutant cooperation, we study the fitness of the double mutant, relative to the fitness of mutants for the cooperative trait alone, that is, *w*_*s*|*c*_=*w*/*w_c_*. Note that when *b = c =* 0, *w*_*s*|*c*_=*w_s_*. The condition for selection to favor additional investments in spite in a mutant cooperative strain is then *w*_*s*|*c*_ > 1, which can be written as

(A8)

The region where this inequality is met and selection for additional spite is favored is illustrated by the solid-line region in Figure 1. Note that when *b = c*= 0, the square brackets equal 1 and eq. A8 simplifies to eq. A5. To identify whether the modifying effect of cooperation enhances selection for spite, we look for parameter regions where *w*_*s*|*c*_ > 1 (solid-line region, Fig. 1) is more permissive of spite evolution than *w_s_* > 1 (dashed-line region, Fig. 1). Specifically, we look for conditions where both inequalities *w*_*s*|*c*_ > 1 and *w_s_* < 1 are met. Some algebra shows this to be the case whenever *w_c_* > 1 (eq. A7), that is, when cooperative investment is present and the mutant strain is at an appreciable frequency and competition is sufficiently global. This effect can be seen graphically in Figure 1; the region favoring spite given cooperation (solid line, Fig. 1) is enlarged with respect to the region favoring spite alone (dashed line, Fig. 1) only in the region favoring cooperation alone (dotted line, Fig. 1).

(A9)

### INTERACTIONS MEDIATED BY CHANGES IN LOCAL FREQUENCY, *p*

One general assumption of our model approach that is worth further examination is the assumption of weak selection, that is, the social traits themselves do not immediately modify the population structure, represented by the local frequency of the focal strain, *p*. However, it is well understood that investments in spite can significantly increase the local frequency of the focal lineage (due to death of competitors), whereas investments in cooperation can decrease the local frequency (due to competition from cheats). To explore the consequences of changing *p* on the value of investment in cooperation, consider that

(A10)

From eq. A10, we see that increasing spite (by increasing *p*) can have a positive effect on the value of investments in cooperation. Conversely,

(A11)

Here, we see that increasing cooperation (if decreases *p* below (*d + k*)/2*k*) can enhance selection for spite.

### VIRULENCE

We now return to the simple fecundity equations (A2) to ask what are the general implications for host virulence of varying proportions *p* of a double mutant lineage competing within a focal host with a wild-type lineage. We make the simplifying assumption that host virulence *v* (additional host mortality) is directly proportional to the average fecundity of pathogens within a focal host *g_l_* (see eq. A2b). This assumption is supported by our virulence assay that measured bacterial density (Fig. 6A). Now we ask, what is the effect on virulence of increasing the proportion of the focal mutant strain?

(A12)

Thus, in the absence of any spiteful investment *(k = d*= 0), we see that increasing the local presence of a cooperative lineage will always increase virulence (Brown et al. 2002; West and Buckling 2003; Harrison et al. 2006; Brown et al. 2009). Conversely, in the absence of any cooperative trait (*b*=*c*= 0), we see that increasing the local presence of a spiteful lineage competing with a nonspiteful lineage will decrease virulence if *p <* (*d + k*)/2*k*, and then increase virulence thereafter. Given the cost of spite is less than the magnitude of its maximal effect (i.e., *d* < *k*), this result implies that virulence will be minimized for intermediate local frequencies of a spiteful lineage (Gardner and West 2004; Inglis et al. 2009). Finally, for the case of a double mutant, we can see from equation (A12) that increasing the local presence of the mutant strain will increase virulence whenever

Therefore, the intermediate minima that are characteristic of spite-mediate virulence (Gardner et al. 2004; Inglis et al. 2009) are lost whenever *b > c + d + k*. Figure 3 illustrates the virulence *v* as a function of *p*, under these three distinct scenarios.
